# Diarylboron‐Based Asymmetric Red‐Emitting Ir(III) Complex for Solution‐Processed Phosphorescent Organic Light‐Emitting Diode with External Quantum Efficiency above 28%

**DOI:** 10.1002/advs.201701067

**Published:** 2018-03-06

**Authors:** Xiaolong Yang, Haoran Guo, Boao Liu, Jiang Zhao, Guijiang Zhou, Zhaoxin Wu, Wai‐Yeung Wong

**Affiliations:** ^1^ MOE Key Laboratory for Nonequilibrium Synthesis and Modulation of Condensed Matter Institute of Chemistry for New Energy Material Department of Chemistry School of Science State Key Laboratory for Mechanical Behavior of Materials Xi'an Jiaotong University Xi'an 710049 P. R. China; ^2^ Key Laboratory for Physical Electronics and Devices of the Ministry of Education Faculty of Electronic and Information Engineering Xi'an Jiaotong University Xi'an 710049 P. R. China; ^3^ Department of Applied Biology and Chemical Technology The Hong Kong Polytechnic University Hung Hom Hong Kong P. R. China

**Keywords:** asymmetric Ir(III) complexes, diarylboron, high efficiency diodes, red phosphor, solution‐processed organic light‐emitting diodes

## Abstract

Organic light‐emitting diodes (OLEDs) are one of the most promising technologies for future displays and lighting. Compared with the blue and green OLEDs that have achieved very high efficiencies by using phosphorescent Ir(III) complexes, the red OLEDs still show relatively low efficiencies because of the lack of high‐performance red‐emitting Ir(III) complexes. Here, three highly efficient asymmetric red‐emitting Ir(III) complexes with two different cyclometalating ligands made by incorporating only one electron‐deficient triarylboron group into the nitrogen heterocyclic ring are reported. These complexes show enhanced photoluminescence quantum yields up to 0.96 and improved electron transporting capacity. In addition, the asymmetric structure can help to improve the solubility of Ir(III) complexes, which is crucial for fabricating OLEDs using the solution method. The photoluminescent and oxidation–reduction properties of these Ir(III) complexes are investigated both experimentally and theoretically. Most importantly, a solution‐processed red OLED achieves extremely high external quantum efficiency, current efficiency, and power efficiency with values of 28.5%, 54.4 cd A^−1^, and 50.1 lm W^−1^, respectively, with very low efficiency roll‐off. Additionally, the related device has a significantly extended operating lifetime compared with the reference device. These results demonstrate that the asymmetric diarylboron‐based Ir(III) complexes have great potential for fabricating high‐performance red OLEDs.

## 1 Introduction

Organic light‐emitting diodes (OLEDs) have been regarded as the most promising candidate for both displays and solid‐state lighting sources.^1^, ^2^, ^3^ In the development of OLEDs, the most important issue is the research on emitting materials because they have crucial influence on the emission colors and efficiencies.^4^, ^5^ Efficient blue, green, orange, and red‐emitting materials are essential to develop high‐performance OLEDs applied to displays and solid‐state lighting sources. Among all the emitting materials developed for OLEDs, phosphorescent transition metal complexes, e.g., Ir(III) and Pt(II) complexes, have drawn great attention due to their capability of harvesting 100% of the both singlet and triplet excitons electrogenerated in devices.^6^, ^7^ Using Ir(III) complexes as emitters, blue, green, and orange OLEDs with external quantum efficiencies (EQEs) over 30% have been reported.^8^, ^9^, ^10^, ^11^, ^12^ However, even based on elaborately designed bipolar host materials and sophisticated device structures, only several most efficient vacuum‐deposited red OLEDs using Ir(III) complexes as emitters show EQEs around 26%.^11^, ^13^, ^14^, ^15^, ^16^ As for the solution‐processed red OLEDs based on Ir(III) complexes, the highest EQEs are less than 22%.^17^, ^18^, ^19^ Compared with the extremely high efficiencies of blue, green, and orange OLEDs, one of the reasons for the inferior efficiencies of these red OLEDs is the low photoluminescence quantum yields (PLQYs) of the emitter. For example, the PLQYs of the widely used red phosphors bis(2‐methyldibenzo‐[*f,h*]‐quinoxaline)Ir(III)(acetylacetonate) [(MDQ)_2_Ir(acac)] and bis(1‐phenylisoquinoline)Ir(III)(acetylacetonate)[(piq)_2_Ir(acac)] are only 0.48 and 0.2, respectively.^20^, ^21^ According to “the energy gap law,”^22^ as the energy gap between the emissive excited state and the ground state decreases, the nonradiative rate constant (*k*_nr_) increases while the radiative rate constant (*k*_r_) decreases. Thus, the red emissive materials with narrow energy gaps tend to display low PLQYs.^23^ However, because the phosphorescence of Ir(III) complexes mainly result from the metal‐to‐ligand charge transfer (MLCT) and ligand‐centered (LC) transitions, the emission properties including the PLQY of an Ir(III) complex are decided by the organic ligands. It means that there is still a chance to improve the photoluminescence quantum yield (PLQY) of an Ir(III) complex by carefully designing the organic ligands.

**Scheme 1:**
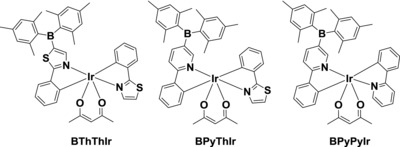
Structural drawing of diarylboron‐based asymmetric Ir(III) complexes.

Recently, we reported a diarylboron‐based red‐emitting Ir(III) complex [bis(5‐(dimesitylboranyl)‐2‐phenylpyridine)Ir(III)(acetylacetonate), **Ir‐B‐1**] showing high PLQY of 0.95 in CH_2_Cl_2_, which is among the highest PLQYs reported for red‐emitting Ir(III) complexes.^24^ The enhanced PLQY could be attributed to the incorporation of electron‐deficient diarylboron group into the pyridine ring, which could increase the mixing of the MLCT state and the triplet ligand‐centered (^3^LC) state of the complex.^25^ Moreover, the electron‐deficient diarylboron group could improve the electron‐transporting ability of the resultant complex.^24^, ^26^ Therefore, a conventional vacuum‐deposited OLED using **Ir‐B‐1** as emitter could show high EQE of 14.7% with low efficiency roll‐off, which is much better than the control device (EQE = 6.0%) based on (MDQ)_2_Ir(acac).^24^ However, **Ir‐B‐1** shows relatively poor solubility in common organic solvents, which makes it unsuitable for fabricating OLEDs with solution‐processed method. Considering the advantages of the solution‐processed method in fabricating large‐area and flexible OLEDs via the low‐cost printing method or roll‐to‐roll coating technique,^27^, ^28^, ^29^ it is in urgent demand to develop efficient red Ir(III) complexes for applications in high‐performance solution‐processed red OLEDs. Therefore, following the design experience of **Ir‐B‐1**, we developed another three diarylboron‐based asymmetric Ir(III) complexes as shown in **Scheme** **1**. Different from the common structures of heteroleptic Ir(III) complexes such as **Ir‐B‐1** in which the two cyclometalating ligands are identical except for the ancillary ligand acetylacetonate (acac), the structures of these three newly synthesized Ir(III) complexes contain two different cyclometalating ligands, i.e., one cyclometalating ligand has a diarylboron group and the other does not. We design these Ir(III) complexes based on the following reason. In addition to the enhancement of the electron‐transporting ability, the incorporation of electron‐deficient diarylboron group into one of the cyclometalating ligands can notably increase the PLQY. The other cyclometalating ligand without diarylboron group can break the symmetric structure of the molecule to improve the solubility. Consequently, these Ir(III) complexes with totally asymmetric structures can show good solubilities in common organic solvents with high PLQYs up to 0.89 in THF. In addition, the PLQY of **BPyThIr** doped 4,4′,4″‐tri(*N*‐carbazolyl)triphenylamine (TCTA) film can be increased to 0.96. Most importantly, the solution‐processed red OLED based on **BPyThIr** shows an extremely high EQE of 28.5%, which is the highest record for the solution‐processed red OLED to date.^17^, ^18^, ^19^ In addition, the operation lifetime of the device using **BPyThIr** as emitter is significantly improved over 30% compared to the device based on (MDQ)_2_Ir(acac).

## 2 Results and Discussion

The syntheses of these diarylboron‐based Ir(III) complexes were similar to the common methods reported for other heteroleptic Ir(III) complexes except the solvents used here were the mixture of THF and H_2_O rather than the conventional used mixture of 2‐ethoxyethanol and H_2_O (see the Supporting Information).^30^ Adopting the one‐pot method, one asymmetrical complex and two symmetric complexes were formed at the same time with acceptable yields. If the two cyclometalating ligands were heated with IrCl_3_ one after another, isolated yields of the asymmetric complex could be further improved. The NMR and mass spectral investigations confirmed the asymmetric structures (see Figures S1 and S2 in the Supporting Information) of these Ir(III) complexes. Their thermal properties were evaluated by the thermogravimetric analysis. The decomposition temperatures (*T*_d_) of these Ir(III) complexes were in the range from 225 to 267 °C (**Table** **1** and Figure S3, Supporting Information), which are high enough for the device fabrication processes and practical usages.

**Table 1:** Photophysical, thermal data, and HOMO/LUMO levels for these diarylboron‐based asymmetric Ir(III) complexes

Complex	λ_abs_ a) [nm] [logε]	λ_em_ [nm] THFa)/filmb)	PLQY THFa)/filmb)	τ_p_ [µs] THFa)/filmb)	*T*_d_ [°C]	HOMO/LUMO [eV]
**BThThIr**	293 (4.21), 353 (4.17), 427 (3.54), 538 (3.40)	623/612	0.75/0.76	1.39/1.82	225	−5.37/−2.96
**BPyThIr**	309 (4.40), 342 (4.44), 427 (3.62), 530 (3.53)	619/602	0.89/0.96	0.93/0.78	262	−5.26/−2.80
**BPyPyIr**	339 (4.57), 432 (3.72), 531 (3.70)	621/607	0.69/0.35	0.93/0.73	267	−5.26/−2.81

^a)^ The λ_abs_ and λ_em_ were measured in THF at a concentration of 10^−5^ m; the PLQYs were measured in degassed THF solution relative to *fac*‐[Ir(ppy)_3_] (PLQY = 0.40); the τ_p_ were recorded in degassed THF solution

^b)^ Measured in Ir(III) complexes doped TCTA films at the doping level of 10 wt%.

**Figure 1:**
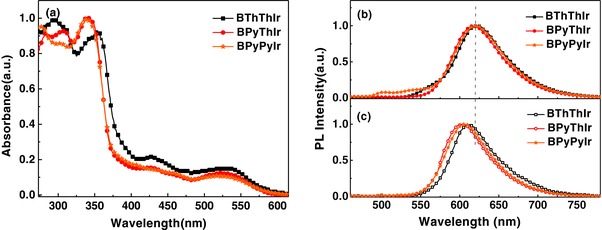
a) Absorption and b) emission spectra of these asymmetric Ir(III) complexes recorded in THF; c) emission spectra of doped TCTA film at the 10 wt% doping level.

**Figure** **1**a shows the absorption spectra of these diarylboron‐based asymmetric Ir(III) complexes in THF solutions. The strong absorption bands in UV region can be ascribed to the spin‐allow singlet ^1^π–π* transition of the two different cyclometalating ligands. Specifically, the absorption peaks from 325 to 360 nm can be assigned to the ^1^π–π* transition of cyclometalating ligands bearing diarylboron group since **BPyThIr** and **BPyPyIr** with the same diarylboron substituted cyclometalating ligand show the same absorption profile around 340 nm. The absorption peaks in higher energy region (<325 nm) should be attributed to the ^1^π–π* transition of cyclometalating ligands without the diarylboron group. The weaker absorption occurring in the visible region can be assigned to the singlet and triplet MLCT transition. The singlet and triplet ligand–ligand charge transfer (LLCT) and LC excited states also contribute to the absorption in the visible region, as confirmed by time‐dependent density functional theory (TD‐DFT) calculations (vide infra). As depicted in Figure 1b and listed in Table 1, these complexes showed red emissions with peaks (λ_em_) around 620 nm in THF solutions. The broad featureless emission profiles in the red region indicate these complexes emit primarily from MLCT states.^30^ Actually, with the polarity of organic solvents increased, the emission peaks were redshifted, and the emission profiles became broad and structureless (Figure S4, Supporting Information), which is similar to the behaviors of other phosphorescent transition metal complexes.^31^, ^32^, ^33^ The notable solvatochromism of these Ir(III) complexes caused by the differences in the dipole moments between the ground (S_0_) and the first triplet excited (T_1_) states indicates strong charge transfer character in their T_1_ state.^31^, ^32^, ^33^ Meanwhile, the different changes of dipole moment in the excited state versus ground state might also be responsible for the weak emission peak in the range of 500–550 nm for **BPyPyIr**.^31^, ^33^ Anyway, very high PLQYs of these complexes were measured up to 0.89 in THF solutions. Compared with those in THF solutions, the emissions of these complexes in TCTA films show hypsochromic shifts (Figure 1c), which are induced by the restricted dipole–dipole relaxation in the rigid matrix, implying the charge redistribution characters between the excited and ground states.^34^ More importantly, the doped TCTA films also showed very high PLQYs, among which the **BPyThIr** doped film could show PLQY up to 0.96. It was interesting that the differences of PLQYs between these Ir(III) complexes in films were much greater than in THF solutions. Therefore, we turn our attention to the morphology of TCTA films doped with these Ir(III) complexes. The atomic force microscope (AFM) test results revealed that the BPyThIr‐based film showed a small surface roughness with the root mean square (Sq) value of 1.69 nm (Figure S5, Supporting Information). In comparison, the BPyPyIr‐based film displayed a large Sq up to 3.40 nm. Unlike the case of the BPyThIr‐based film, there existed obviously discontinuous regions in the BPyPyIr‐based film, indicating the emitter aggregation induced phase separation to some extent. Therefore, the AFM images implied that the emitter aggregation behavior was more severe in the BPyPyIr‐based film, which might cause triplet–triplet annihilation effect, leading to the lowest PLQY of the BPyPyIr‐based film. On the whole, the incorporation of diarylboron moieties has greatly influenced the emission properties of these Ir(III) complexes. Remarkable emission redshifts of these diarylboron‐based asymmetric Ir(III) complexes with significantly improved PLQYs have been observed, since the related symmetric Ir(III) complexes without the diarylboron substituent usually show green or green‐yellow emissions with PLQYs less than 0.4.^35^, ^36^ In detail, when introducing the diarylboron unit to the thiazole ring, the resulted complex **BThThIr** displayed longer emission wavelength both in the THF solution and TCTA film, and also its emission lifetimes (τ_p_) were the longest (Table 1 and Figure S6, Supporting Information). With the diarylboron unit attached on the pyridine ring, **BPyThIr** exhibited the improved PLQY with reduced τ_p_ compared with **BThThIr**. Replacing the 2‐phenylthizole ligand in **BPyThIr** with the 2‐phenylpyridine ligand reduced the PLQYs of **BPyPyIr** both in THF solution and TCTA film. These observations demonstrate that the diarylboron unit substituted ligands have dominated effect on the emission properties, while the ligand without diarylboron unit can fine tune the emission wavelengths and PLQYs. Anyhow, the very high PLQYs endow these diarylboron‐based asymmetric Ir(III) complexes with the great potential to fabricate high‐performance red OLEDs.

**Figure 2:**
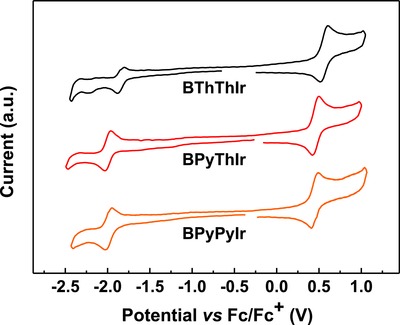
Cyclic voltammogram of these diarylboron‐based asymmetric Ir(III) complexes.

As shown in **Figure** **2**, during the cyclic voltammetric scan in CH_3_CN solutions, these Ir(III) complexes could show reversible metal‐related oxidation waves and reversible diarylboron‐related reduction waves. Interestingly, **BPyThIr** and **BPyPyIr** displayed similar oxidation and reduction behaviors, indicating that they possess similar highest occupied molecular orbital (HOMO)/lowest unoccupied molecular orbital (LUMO) levels (Table 1). Given the fact that **BPyThIr** and **BPyPyIr** contain the same diarylboron substituted cyclometalating ligand, this result implies that the diarylboron substituted cyclometalating ligand exerts dominated influence on the HOMO/LUMO of these complexes. Therefore, with the diarylboron group attached on the thiazole unit, **BThThIr** indeed showed different oxidation and reduction behaviors in CH_3_CN solution compared with **BPyThIr**, i.e., both the HOMO/LUMO levels of **BThThIr** (−5.37/−2.96 eV) were lower than those of **BPyThIr** (−5.26/−2.80 eV). Compared with the symmetric Ir(III) complex **Ir‐B‐1** (HOMO/LUMO levels: −5.22/−2.60 eV), the asymmetric Ir(III) complex **BPyPyIr** shows a similar HOMO level of −5.26 eV but a much deeper LUMO level of −2.81 eV. This may be caused by the fact that a competition in the MLCT process (from the Ir center to the diarylboron moiety) exists in the symmetric Ir(III) complex **Ir‐B‐1** between the two diarylboron substituted cyclometalating ligands, but this competition is almost absent in **BPyPyIr**. Due to the asymmetric structure, the only one diarylboron substituted cyclometalating ligand in **BPyPyIr** will be the sole destination to accept the electron transferred from the Ir center, making **BPyPyIr** easier to be reduced. Therefore, one of the advantages by designing the diarylboron‐based asymmetric Ir(III) complexes is to further lower the LUMO levels, which is beneficial to the electron‐injection/transporting process in OLEDs.^25^, ^37^

**Figure 3:**
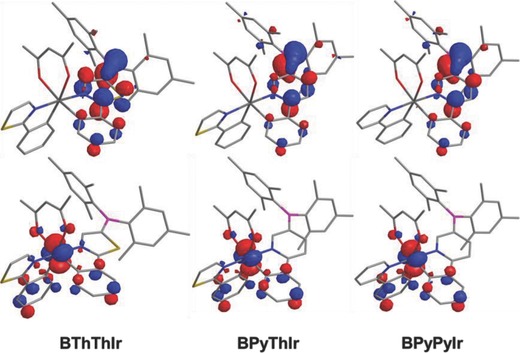
Contour plots of HOMO (bottom) and LUMO (top) of these diarylboron‐based asymmetric Ir(III) complexes.

To gain insight into these photophysical and electrochemical properties, we performed the DFT and TD‐DFT calculations. The calculated phosphorescent emission peaks for **BThThIr**, **BPyThIr,** and **BPyPyIr** (λ_em_^cal.^ = 634, 590, 599 nm, respectively) are in good agreement with the trend of the experimentally observed peaks (λ_em_ = 612, 602, 607 nm, respectively, for **BThThIr**, **BPyThIr,** and **BPyPyIr** in doped films). As shown in **Figure** **3** and Figure S7 (Supporting Information), besides the almost equally contribution from the chelated phenyl fragments of the cyclometalating ligand with and without diarylboron group (15.1–17.1% and 14.9–17.0%, respectively) to the HOMOs of **BThThIr**, **BPyThIr**, and **BPyPyIr**, the d_π_ orbitals of the Ir center in asymmetric Ir(III) complexes make significant contribution (51.4–52.5%) to the HOMOs of these complexes. The LUMOs of these Ir(III) complexes show predominant (more than 92%) distribution over the diarylboron substituted cyclometalating ligand. Specifically, the LUMOs consist of a considerable contribution from the boron atoms (14.5–19.5%). As listed in **Table** **2**, due to large contributions (86.0–97.6%) from HOMO→LUMO transition to both the lowest singlet transition (S_0_→S_1_) and the lowest triplet transition (S_0_→T_1_), all the S_0_→T_1_ transition for these complexes can be assigned to an MLCT character mixed with some contribution from LLCT and ππ* features. These assignments can also be confirmed by the charge density difference for both S_1_ and T_1_ excited states of these Ir(III) complexes (Figure S8, Supporting Information). Therefore, it is reasonable that the diarylboron substituted cyclometalating ligand shows dominant influence on the molecular orbitals, and thereby on the photophysical and electrochemical properties of these complexes.

**Table 2:** Theoretical calculation results for these diarylboron‐based asymmetric Ir(III) complexes

Complexa)	State	λ_cal_ [nm]	*f*	Composition	Assignments
**BThThIr**	S_1_ T_1_	565 634	0.0790	H→L [97.6%] H→L [86.0%]	MLCT/LLCT/ππ* MLCT/LLCT/ππ*
**BPyThIr**	S_1_ T_1_	544 590	0.0659	H→L [97.1%] H→L [91.7%]	MLCT/LLCT/ππ* MLCT/LLCT/ππ*
**BPyPyIr**	S_1_ T_1_	552 599	0.0602	H→L [97.5%] H→L [92.4%]	MLCT/LLCT/ππ* MLCT/LLCT/ππ*

^a)^ H→L represents the HOMO to LUMO transition. *f* stands for oscillator strength.

**Figure 4:**
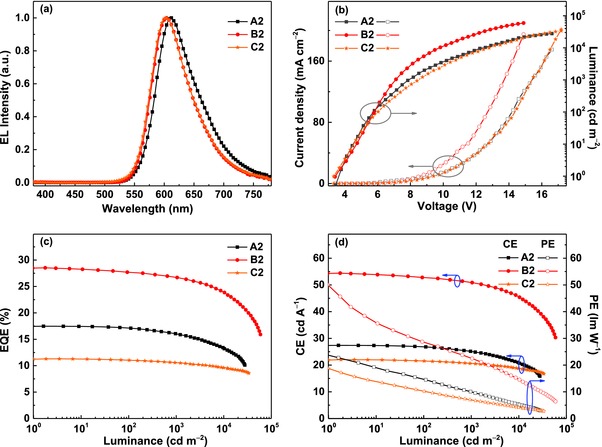
EL characteristics of OLEDs based on **BThThIr**, **BPyThIr**, and **BPyPyIr**: a) EL spectra, b) *J–V–L* characteristics, c) curves of EQE versus luminance, and d) curves of CE and PE versus luminance.

**Figure 5:**
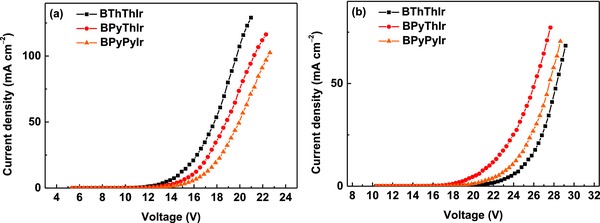
The current density–voltage (*J–V*) curves for a) hole‐only and b) electron‐only devices based on these complexes.

Considering the high PLQYs and good solubility of these complexes, solution‐processed OLEDs were fabricated with the conventional structure of indium tin oxide (ITO)/PEDOT:PSS (45 nm)/*x* wt% **Ir emitter**:TCTA (30 nm)/TPBI (45 nm)/LiF (1 nm)/Al (100 nm) (Figure S9, Supporting Information). We used poly(ethylenedioxythiophene):poly(styrenesulfonate) (PEDOT:PSS) to inject and transport holes to the emissive layer. The emissive layer consisted of the host TCTA and these Ir(III) complexes. We also used 1,3,5‐tris(*N*‐phenylbenzimidazole‐2‐yl)benzene (TPBI) as the electron‐transporting layer, and LiF as the electron‐injection layer. Only the doping level was optimized to excavate the electroluminescence (EL) potential of these Ir(III) complexes (i.e., 6, 10, and 14 wt% of **BThThIr**, **BPyThIr**, and **BPyPyIr** in TCTA for devices **A1**‐**3**, **B1**‐**3**, and **C1**‐**3**, respectively). The detailed energy levels and the molecular structures of the materials used in the fabricated OLEDs are shown in Figure S9 (Supporting Information). All these devices displayed red emissions with EL profiles similar to the corresponding PL spectra in doped TCTA films, and no emission from TCTA (≈390 nm) was detected, indicating the complete energy transfer from the host to the emitter. Among these devices, the devices with 10 wt% doped complexes showed the best performances. Their EL spectra, current density(*J*)–voltage(*V*)–luminance (*L*) curves, and efficiencies versus luminance plots are depicted in **Figure** **4**. The detailed EL data are summarized in **Table** **3**. The performance of other devices is given in the Supporting Information (Figures S10 and S11 and Table S1, Supporting Information). As listed in Table 3, device **A2** based on **BThThIr** showed a turn‐on voltage of 3.5 V with the maximum luminance (*L*_max_), EQE, current efficiency (CE), and power efficiency (PE) of 27 756 cd m^−2^, 17.5%, 27.4 cd A^−1^, and 24.9 lm W^−1^, respectively, which have already outperformed the efficiencies of the vacuum‐deposited device based on the symmetric Ir(III) complex **Ir‐B‐1** (14.7%, 21.4 cd A^−1^, and 22.2 lm W^−1^).^24^ Unexpectedly, device **B2** based on **BPyThIr** exhibited extremely high peak EQE, CE, and PE of 28.5%, 54.4 cd A^−1^, and 50.1 lm W^−1^, respectively, which outstandingly represents for the highest efficiencies for Ir(III) complex‐based red OLEDs fabricated using solution‐processed or vacuum‐deposited method.^11^, ^13^, ^14^, ^15^, ^16^, ^17^, ^18^, ^19^ The remarkable electroluminescent performance of **BPyThIr** could be attributed to the suitable HOMO/LUMO levels, very high PLQY ( = 0.96) as well as good film morphology (Sq = 1.69 nm) of doped TCTA film. Besides, the transporting behaviors of these Ir(III) complexes were investigated by fabricating hole and electron‐only devices with structures of ITO/MoO_3_ (3 nm)/PEDOT:PSS (20 nm)/Ir(III) complex (40 nm)/PEDOT:PSS (20 nm)/MoO_3_ (3 nm)/Al (100 nm) and ITO/LiF (3 nm)/Ir(III) complex (40 nm)/LiF (3 nm)/Al (100 nm), respectively. As shown in **Figure** **5**, under the same driving voltage, the current densities of holy‐only devices are in the order of **BThThIr** > **BPyThIr** > **BPyPyIr**, suggesting that the hole transporting ability of **BPyThIr** is in the middle among that of these three Ir(III) complexes. However, the much higher current density of the electron‐only device based on **BPyThIr** than the other two indicates that **BPyThIr** can show better electron transporting ability, leading to more balanced hole and electron transporting characteristics within the device. Therefore, the more balanced charge transporting ability of **BPyThIr** should also make significant contribution to improve the device efficiencies.^38^ Furthermore, even at high luminance of 1000 cd m^−2^, the EQE and CE of device **B2** still remained as high as 26.7% and 50.9 cd A^−1^, respectively. It should be noted that this extraordinary device performance was achieved with a conventional device structure using the common unipolar host TCTA. This implies that **BPyThIr** has the great potential for practical application in developing more efficient red OLEDs with optimized host materials and device structures. Importantly, the device can be conveniently fabricated using the low‐cost solution‐processed method. Because of similar energy levels of **BPyPyIr** to those of **BPyThIr** (Table 1), the EL profile and turn‐on voltage of device **C2** were similar to those of device **B2**. However, due to the low PLQY (=0.35), very poor film quality (Sq = 3.40 nm) of **BPyPyIr** doped TCTA film, as well as the unbalanced charge transporting properties of **BPyPyIr**, device **C2** displayed inferior performance with peak EQE, CE, and PE of 11.3%, 22.0 cd A^−1^, and 18.8 lm W^−1^, respectively. Nevertheless, device **C2** showed very low efficiency roll‐off even at high luminance of 1000 cd m^−2^.

**Table 3:** EL data for devices **A2**, **B2**, and **C2**

	Emitter	λ_EL_^max^ [nm]	Va) [V]	*L*_max_ [cd m^−2^]	EQEb) [%]	CEb) [cd A^−1^]	PEb) [lm W^−1^]	CIE (*x*, *y*)
**A2**	**BThThIr** (10 wt%)	612	3.5/7.8/12.3	27756	17.5/16.1	27.4/25.2	24.9/10.1	(0.62, 0.38)
**B2**	**BPyThIr** (10 wt%)	604	3.4/7.1/9.8	59154	28.5/26.7	54.4/50.9	50.1/23.0	(0.61, 0.39)
**C2**	**BPyPyIr** (10 wt%)	604	3.3/8.4/12.8	33659	11.3/10.6	22.0/20.6	18.8/7.0	(0.60, 0.40)

^a)^ Driving voltages (V) in the order of at 1, 1000, and 10 000 cd m^−2^, respectively

^b)^ EQE, CE, and PE in the order of the maximum value and at 1000 cd m^−2^.

**Figure 6:**
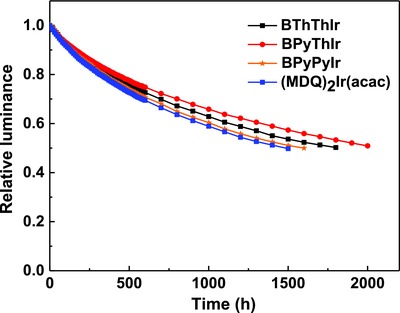
The operating lifetimes for OLEDs based on **BThThIr**, **BPyThIr**, **BPyPyIr**, and (MDQ)_2_Ir(acac).

The operation stability of the unsealed devices based on these diarylboron‐based asymmetric Ir(III) complexes was tested under the N_2_ atmosphere. For comparison, a control device based on (MDQ)_2_Ir(acac) was also fabricated with the same device structure. **Figure** **6** shows the relative luminance versus time for these devices operated with the initial luminance (*L*_0_) of 1000 cd m^−2^. The operation lifetime value at 50% initial luminance, LT_50_, for the control device based on (MDQ)_2_Ir(acac) is about 1500 h, while the LT_50_ for devices based on our newly synthesized Ir(III) complexes is over 1500 h. Especially, the LT_50_ for the device based on **BPyThIr** is about 2000 h, indicating the much higher device stability of using **BPyThIr** as the emitter than using the widely used (MDQ)_2_Ir(acac).

## 3 Conclusion

In summary, we report three red‐emitting asymmetric Ir(III) complexes and demonstrate that the introduction of the diarylboron group into one of the cyclometalating ligands can also significantly improve the PLQYs and lower the LUMO levels of the resultant Ir(III) complexes, which are beneficial to the EL performance. Most importantly, the solution‐processed red OLED using the common unipolar host TCTA and the emitter **BPyThIr** can achieve extremely high peak EQE, CE, and PE of 28.5%, 54.4 cd A^−1^, and 50.1 lm W^−1^, respectively, with very low efficiency roll‐off even at high luminance of 1000 cd m^−2^. In addition, the lifetime value at 50% initial luminance for the device based on **BPyThIr** is about 2000 h. These results represent one of the best performances for Ir(III) complex‐based red OLEDs reported so far, indicating that **BPyThIr** is very promising for practical application in red OLEDs. This work shows the great potential to design diarylboron‐based Ir(III) complexes with an asymmetric molecular structure for highly efficient OLEDs.

## Conflict of Interest

The authors declare no conflict of interest.

## Supporting information

Supplementary
