# Supplementary material for: Diarylboron‐Based Asymmetric Red‐Emitting Ir(III) Complex for Solution‐Processed Phosphorescent Organic Light‐Emitting Diode with External Quantum Efficiency above 28%
Source: Adv Sci (Weinh). 2018 Mar 6;5(5):1701067. doi: 10.1002/advs.201701067 (PMC5979779; doi:10.1002/advs.201701067)
Supplement: Supplementary file 1 — Supplementary [file ADVS-5-1701067-s001.pdf]

Copyright WILEY-VCH Verlag GmbH & Co. KGaA, 69469 Weinheim, Germany, 2018.

ADVANCED SCIENCE
Open Access

Supporting Information

for *Adv. Sci.,* DOI: 10.1002/advs.201701067

Diarylboron-Based Asymmetric Red-Emitting Ir(III) Complex for Solution-Processed Phosphorescent Organic Light-Emitting Diode with External Quantum Efficiency above 28%

Xiaolong Yang, Haoran Guo, Boao Liu, Jiang Zhao, Guijiang Zhou, Zhaoxin Wu,* and Wai-Yeung Wong**

Supporting Information

Diarylboron-Based Asymmetric Red-Emitting Ir(III) Complex for Solution-Processed Phosphorescent Organic Light-Emitting Diode with External Quantum Efficiency Above 28%

Xiaolong Yang, Haoran Guo, Boao Liu, Jiang Zhao, Guijiang Zhou, Zhaoxin Wu* and Wai-Yeung Wong**

General Experimental Information

Commercially available reagents were used directly without further purification. All reactions were carried out under a N_2 atmosphere. ^1H NMR and ^{13}C NMR spectra were measured with a Bruker Avance 400 MHz spectrometer in $CDCl_3$. Elemental analyses were measured on a Flash EA 1112 elemental analyzer. Mass spectral (MS) measurements were performed on a micrOTOF-Q II mass spectroscopy. The thermal gravimetric analysis (TGA) was measured with a NETZSCH STA 409C instrument under N_2 at a heating rate of 20 K min^{-1}. The atomic force microscopy (AFM) images were recorded on a NT-MDT Atomic Force Microscope NEXT. UV-vis absorption spectra were recorded on a Shimadzu UV-2250 spectrophotometer in THF at room temperature. Photoluminescent spectra and lifetimes of these complexes were tested on an Edinburgh Instruments Ltd (FLSP920) fluorescence spectrophotometer. The solution PLQYs were determined in degassed THF solutions at room temperature against *fac*-[Ir(ppy)$_3$] standard (PLQY = 0.4). The PLQYs of doped films were determined with an integrating sphere. Cyclic voltammetry investigations were performed on the Princeton Applied Research (PARSTAT 2273, Advanced Electrochemical System) equipment in CH_3CN solutions containing *n*-Bu_4NPF_6 (0.1 M) as the supporting electrolyte at the scan rate of 100 mV s^{-1}, and calibrated with ferrocene/ferrocenium (Fc/Fc^+) couple. The HOMO and LUMO energy levels were calculated using the oxidation potential ($E_{ox}^{1/2}$) and reduction potential ($E_{red}^{1/2}$) according to the equations $E_{HOMO} = -(E_{ox}^{1/2} + 4.8)$ eV and $E_{LUMO} = -(E_{red}^{1/2} + 4.8)$ eV.

Synthesis of 5-(dimesitylboryl)-2-phenylthiazole (BTh)

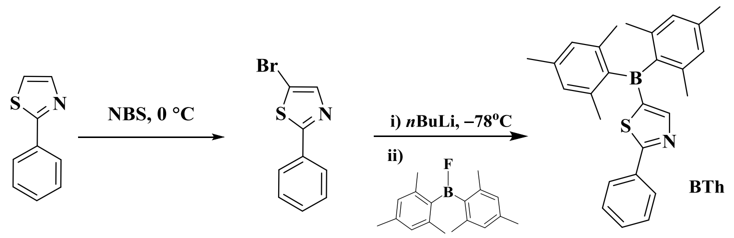

5-bromo-2-phenylthiazole To a solution of 2-phenylthiazole (2.03 g, 12.6 mmol) in $CHCl_3$ (30 mL) and CH_3COOH (2 mL) at 0 °C, NBS (2.36 g, 13.2 mmol) was added very slowly. Then the mixture was allowed to warm to room temperature and stirred for 16 h. After washing with water, the organic layers were combined, dried over anhydrous Mg_2SO_4 and concentrated. The residual was purified on a silica column using a mixture of petroleum ether and CH_2Cl_2 (v/v, 1:1) as eluent to give 2.06 g white solid in 68.4 % yield. ^{1}H NMR (400 MHz, $CDCl_3$): δ (ppm) 7.86–7.85 (m, 2H), 7.74 (s, 1H), 7.44–7.43 (m, 3H); ^{13}C NMR (100 MHz, $CDCl_3$): δ (ppm) 169.59, 144.86, 133.11, 130.45, 129.08, 126.23, 108.53.

5-(dimesitylboryl)-2-phenylthiazole (BTh) A solution of 5-bromo-2-phenylthiazole (0.81 g, 3.4 mmol) in dry ethyl ether (30 mL) was cooled to –78 °C under the nitrogen atmosphere, followed by addition of *n*-BuLi (1.4 mL, 3.5 mmol). The mixture was stirred for 30 min at –78 °C and then fluorodimesitylborane (1.0 g, purity, 90 %) was added. The reaction solution was warmed to room temperature and stirred for 4 h. After adding water (10 mL), the mixture was extracted with CH_2Cl_2 several times. The organic layers were combined, dried over anhydrous Mg_2SO_4 and concentrated. The residual was purified on a silica column using a mixture of petroleum ether and

CH_2Cl_2 (v/v, 5:2) as eluent to give 0.45 g white solid in 32.3 % yield. ^{1}H NMR (400 MHz, $CDCl_3$): δ (ppm) 8.04–7.01 (m, 3H), 7.44–7.43 (m, 3H), 6.86 (s, 4H), 2.32 (s, 6H), 2.15 (s, 12H); ^{13}C NMR (100 MHz, $CDCl_3$): δ (ppm) 178.06, 155.53, 140.63, 139.10, 133.46, 130.76, 128.98, 128.38, 126.99, 23.45, 21.23.

BPy was synthesized following our previously reported method.[1]

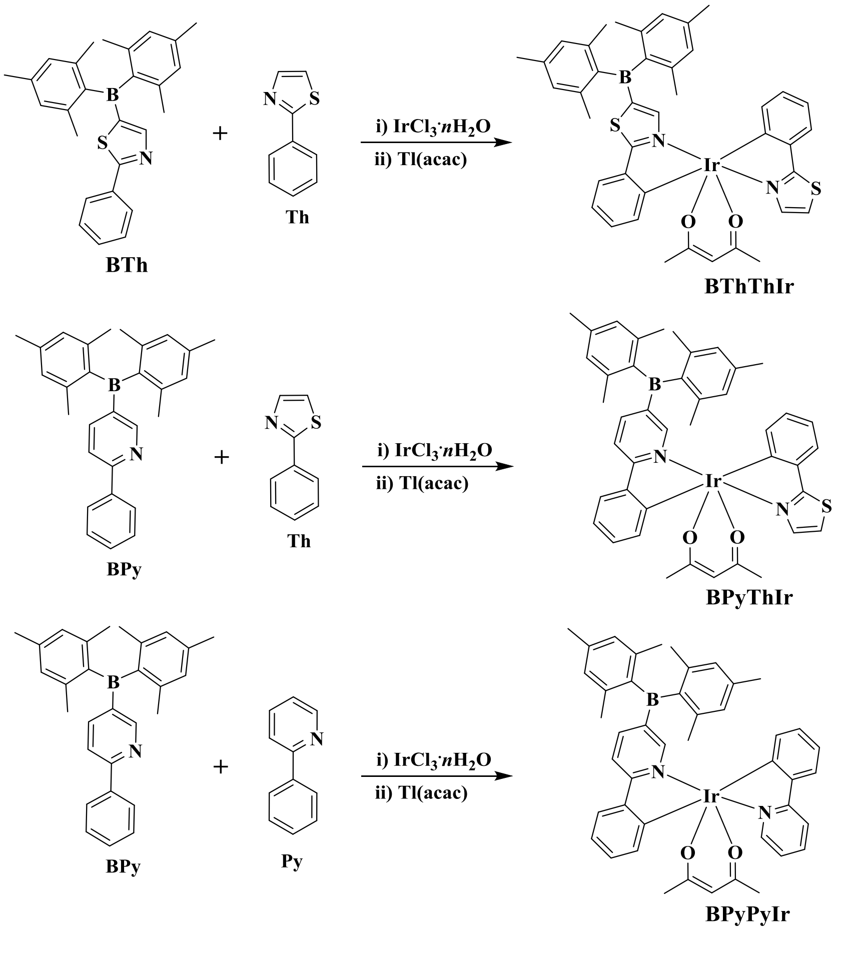

General procedure for synthesizing the Ir(III) complexes.

Under a N_2 atmosphere, ligand with boric moiety (**BTh** or **BPy**) (1.0 eqv.), ligand without boric moiety (2-phenylthiazole (**Th**), 2-phenylpyridine (**Py**)) (1.0 eqv.) and $IrCl_3$•nH_2O (1.0 eqv.) were added to a mixture of tetrahydrofuran and water (3:1, v/v)

(25 mL). The reaction mixture was heated to 110 °C for ~16 h with stirring. After cooling to room temperature, water (10 mL) was added to the reaction mixture. The mixture was extracted with CH_2Cl_2 several times. The organic layers were combined, dried over anhydrous sodium sulfate and concentrated. Without purification, together with thallium(I) acetylacetonate (1.0 eqv.), the residue were dissolved in CH_2Cl_2 (20 mL) under a N_2 atmosphere. After stirring for ~16 h at room temperature, the solvent was removed under reduced pressure and the residue was purified on preparative TLC plates to give the demanded complexes.

BThThIr Red solid (17.5 %). ^{1}H NMR (400 MHz, $CDCl_3$): δ (ppm) 7.72 (s, 1H), 7.67 (d, *J* = 3.2 Hz, 1H), 7.49–7.46 (m, 2H), 7.35 (d, *J* = 3.2 Hz, 1H), 6.87 (s, 4H), 6.83–6.66 (m, 4H), 6.33 (d, *J* = 7.6 Hz, 1H), 6.30 (d, *J* = 7.2 Hz, 1H), 5.22 (s, 1H), 2.31 (s, 6H), 2.22 (s, 12H), 1.80 (s, 3H), 1.55 (s, 3H); ^{13}C NMR (100 MHz, $CDCl_3$): δ (ppm) 185.15, 184.79, 152.07, 148.01, 145.21, 141.20, 140.87, 140.80, 139.41, 139.29, 134.11, 133.88, 130.27, 129.48, 128.44, 125.49, 124.12, 121.05, 120.83, 116.00, 100.50, 28.27, 28.13, 22.68, 21.22. TOF-MS (*m/z*): 883 $[M+Na]^+$. Elemental analysis calcd (%) for $C_{41}H_{40}BIrN_2O_2S_2$: C 57.27, H 4.69, N 3.26; found: C 57.16, H 4.51, N 3.19.

BPyThIr Red solid (20.0 %). ^{1}H NMR (400 MHz, $CDCl_3$): δ (ppm) 8.53 (s, 1H), 7.79 (d, *J* = 8.0 Hz, 1H), 7.73 (d, *J* = 8.0 Hz, 1H), 7.63 (d, *J* = 7.2 Hz, 1H), 7.58 (d, *J* = 7.2 Hz, 1H), 7.46 (d, *J* = 7.2 Hz, 1H), 7.34 (d, *J* = 3.6 Hz, 1H), 6.83–7.75 (m, 6H), 6.72 (t, *J* = 7.6 Hz, 1H), 6.64 (t, *J* = 7.6 Hz, 1H), 6.30 (t, *J* = 7.6 Hz, 2H), 5.11 (s, 1H), 2.30 (s, 6H), 2.07 (s, 12H), 1.79 (s, 3H), 1.28 (s, 3H); ^{13}C NMR (100 MHz, $CDCl_3$): δ (ppm)

184.91, 184.27, 178.13, 171.39, 156.14, 149.88, 146.10, 144.45, 144.14, 141.29, 140.81, 139.52, 139.20, 133.73, 133.49, 129.90, 129.35, 128.49, 124.09, 120.73, 117.61, 115.94, 100.44, 28.33, 27.97, 23.50, 21.23. TOF-MS (*m/z*): 855 $[M+H]^+$. Elemental analysis calcd (%) for $C_{43}H_{42}BIrN_2O_2S$: C 60.48, H 4.96, N 3.28; found: C 60.43, H 4.52, N 3.22.

BPyPyIr Red solid (16.8 %). ^{1}H NMR (400 MHz, $CDCl_3$): δ (ppm) 8.52–8.49 (m, 2H), 7.81 (t, *J* = 8.0 Hz, 2H), 7.79–7.69 (m, 2H), 7.58 (d, *J* = 8.0 Hz, 1H), 7.51 (d, *J* = 7.6 Hz, 1H), 7.11 (t, *J* = 6.4 Hz, 1H), 6.83 (s, 4H), 6.79 (t, *J* = 7.6 Hz, 2H), 6.68 (t, *J* = 7.6 Hz, 2H), 6.27 (d, *J* = 7.6 Hz, 2H), 5.09 (s, 1H), 2.30 (s, 6H), 2.07 (s, 12H), 1.78 (s, 3H), 1.24 (s, 3H); ^{13}C NMR (100 MHz, $CDCl_3$): δ (ppm) 185.04, 183.92, 171.00, 168.53, 156.06, 150.62, 148.12, 147.60, 144.71, 144.35, 144.26, 140.80, 139.21, 136.74, 133.30, 133.19, 129.82, 129.08, 128.49, 124.94, 123.72, 121.41, 120.72, 120.67, 118.30, 117.64, 100.57, 28.54, 28.03, 23.47, 21.23. TOF-MS (*m/z*): 849 $[M+H]^+$. Elemental analysis calcd (%) for $C_{45}H_{44}BIrN_2O_2$: C 63.75, H 5.23, N 3.30; found: C 63.71, H 5.26, N 3.19.

We also find a way to improved the yield of the asymmetric products. Different from the above mentioned method that the cyclometalating ligands were heated with $IrCl_3$ at the same time, the second cyclometalating ligand was added into the reaction mixture after heating the $IrCl_3$ with the first cyclometalating ligand about three hours later, the rest operation was the same as the method described above. Finally, the yield of **BPyThIr** could be improved to 38.6%. Anyway, the one-pot method

described in this paper is vey convenient to synthesize more useful complexes with acceptable yields within a short time.

Theoretical Computation. Density functional theory (DFT) calculations were performed for all these Ir(III) complexes. Non-metal atoms of C, H, N and O were described by the all-electron basis set of B3LYP/6-31G. The effective core potentials with a B3LYP/LanL2DZ basis set was used for Ir atoms.[2, 3] The excitation behaviors of the complexes were computed by time-dependent density functional theory (TD-DFT) method based on optimized geometries at the ground states. All calculations were carried out by using the Gaussian 09 program.[4]

OLED Fabrication and Measurements. The ITO glass substrates were pre-cleaned and exposed to ultraviolet-ozone for ca. 10 min. As for the PEDOT:PSS was spin-coated on the surface of ITO glass substrates to form a hole-injection layer and annealed at 120 °C for 30 min in the air. Then, the chloroform solution of emitters in the host was spin-coated on surface of PEDOT:PSS layer to form the emission layer. The obtained ITO chip was dried at 60 °C for 10 min before it was transferred to the deposition system to deposit other active layers, i.e., electron-transporting layer, electron-injection layer and Al cathode. The EL spectra were measured with a PR650 spectra colorimeter. The driving voltages and efficiencies of the devices were measured with the Keithley 2602 and Source Meter. The efficiency and spectral measurements were carried out under ambient conditions. The unsealed devices stability were tested under the nitrogen atomosphere.

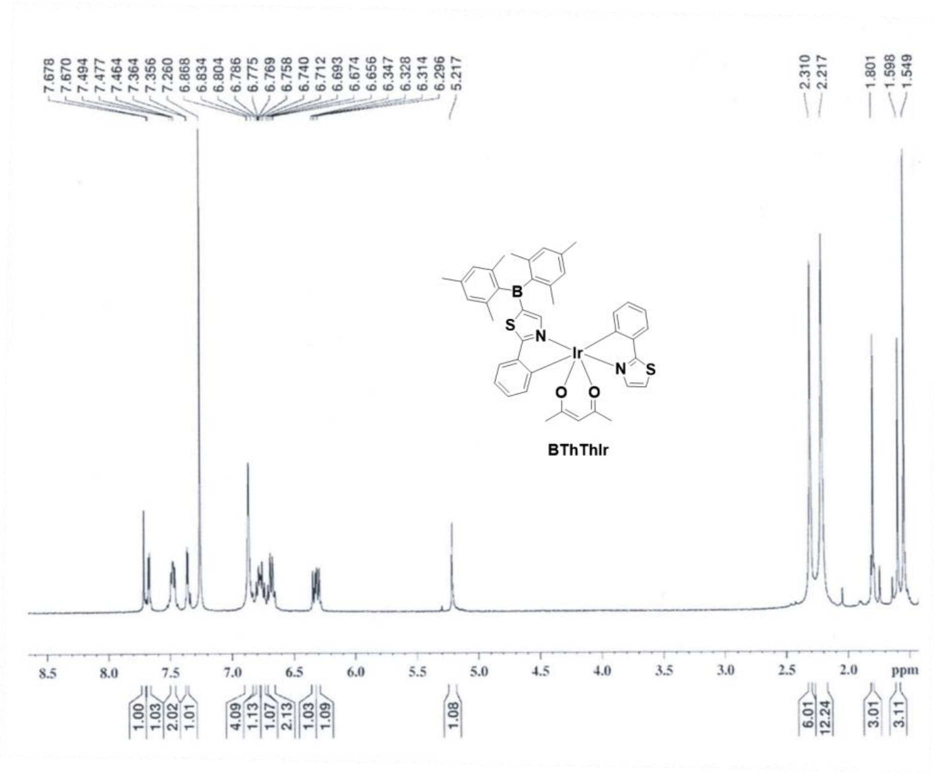
7.678
7.670
7.494
7.477
7.464
7.364
7.356
7.260
6.868
6.834
6.804
6.786
6.775
6.769
6.758
6.740
6.712
6.693
6.674
6.656
6.347
6.328
6.314
6.296
5.217
2.310
2.217
1.801
1.598
1.549
BThThIr
ppm
1.00
1.03
2.02
1.01
4.09
1.13
1.07
2.13
1.03
1.09
1.08
6.01
12.24
3.01
3.11

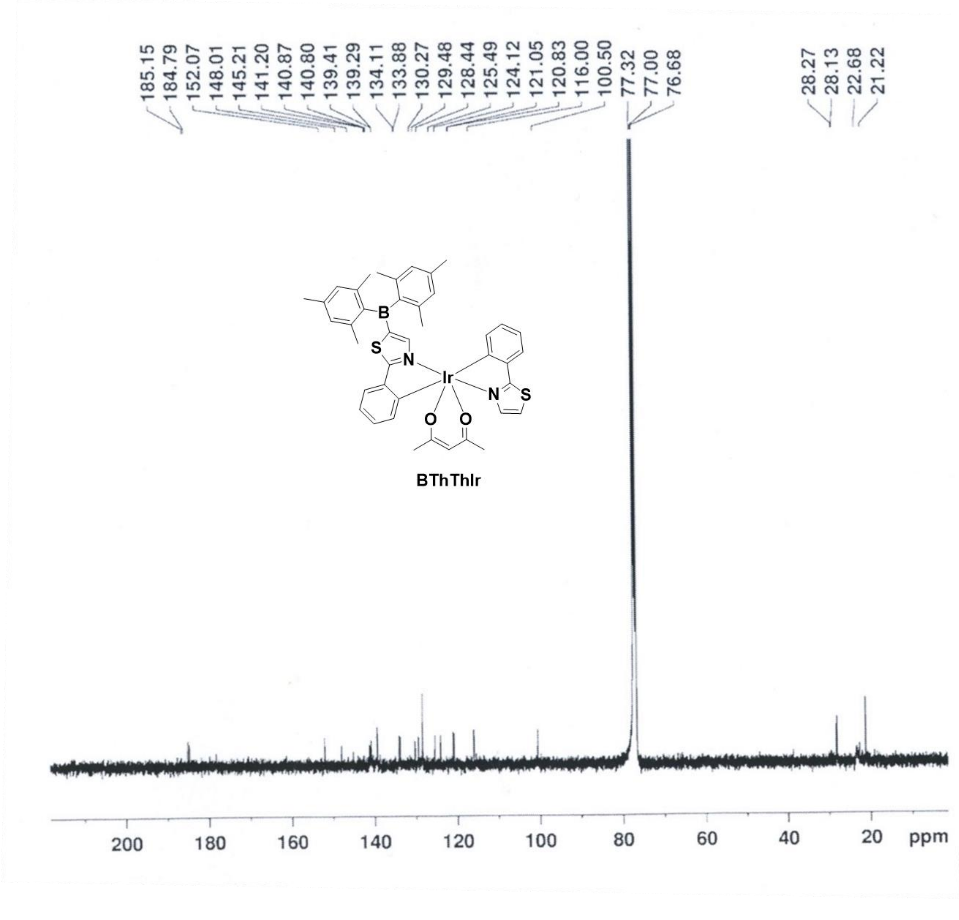
185.15
184.79
152.07
148.01
145.21
141.20
140.87
140.80
139.41
139.29
134.11
133.88
130.27
129.48
128.44
125.49
124.12
121.05
120.83
116.00
100.50
77.32
77.00
76.68
28.27
28.13
22.68
21.22
BThThIr
ppm

Figure S1. NMR spectra of these Ir(III) complexes.

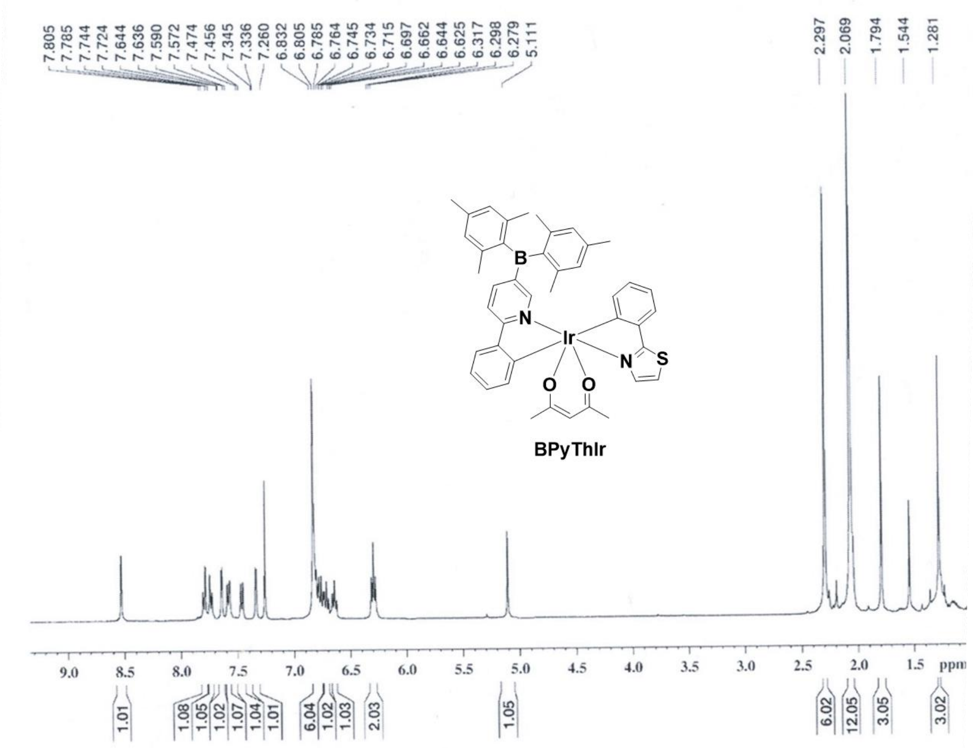

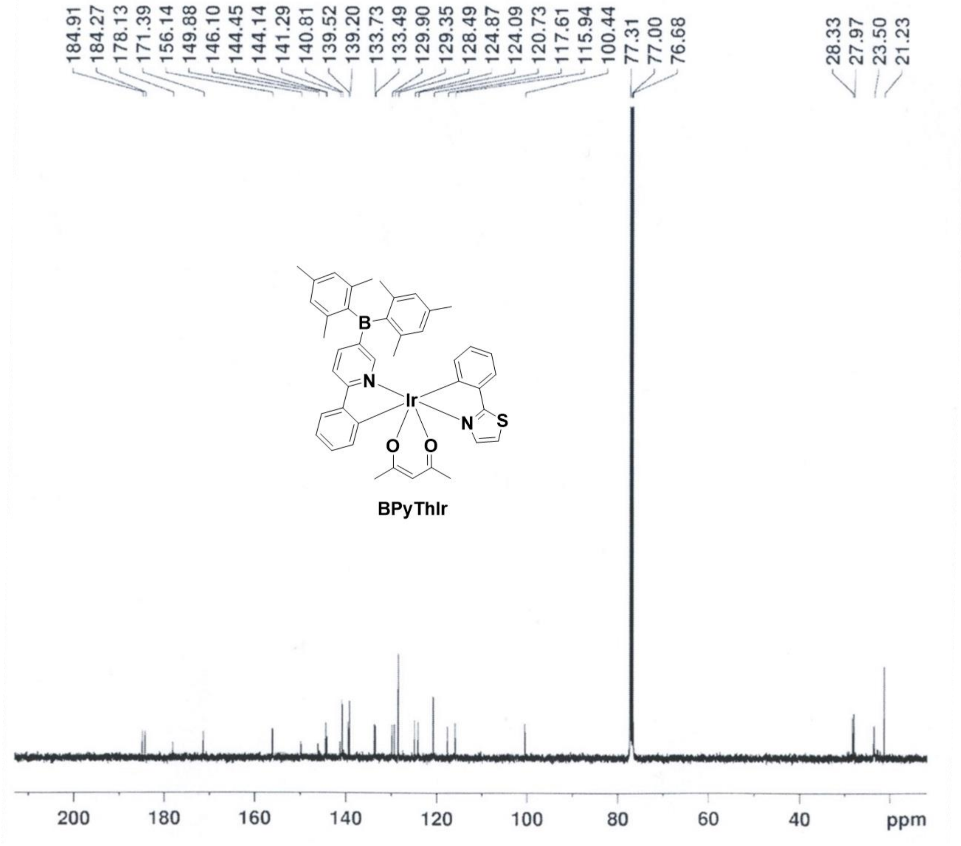

Figure S1. Continued.

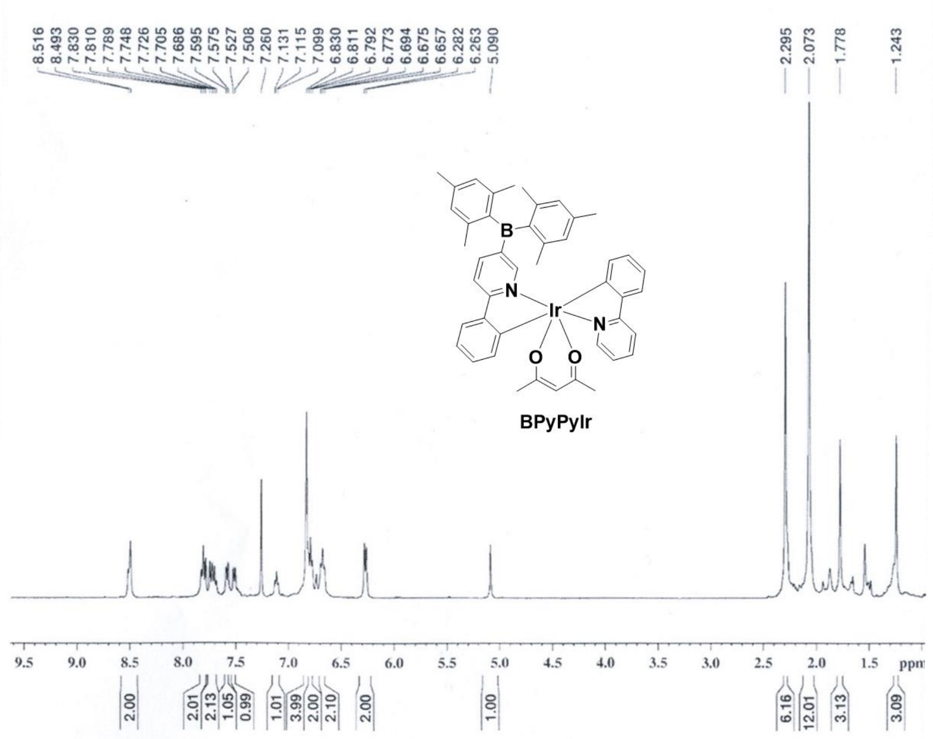

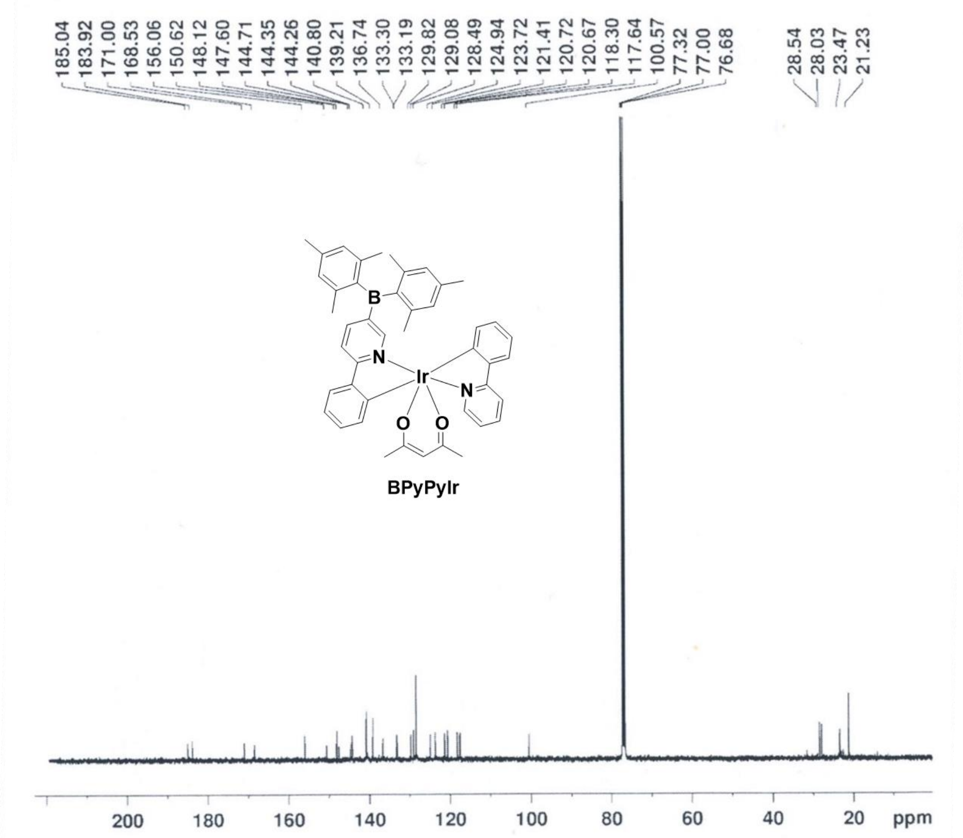

Figure S1. Continued.

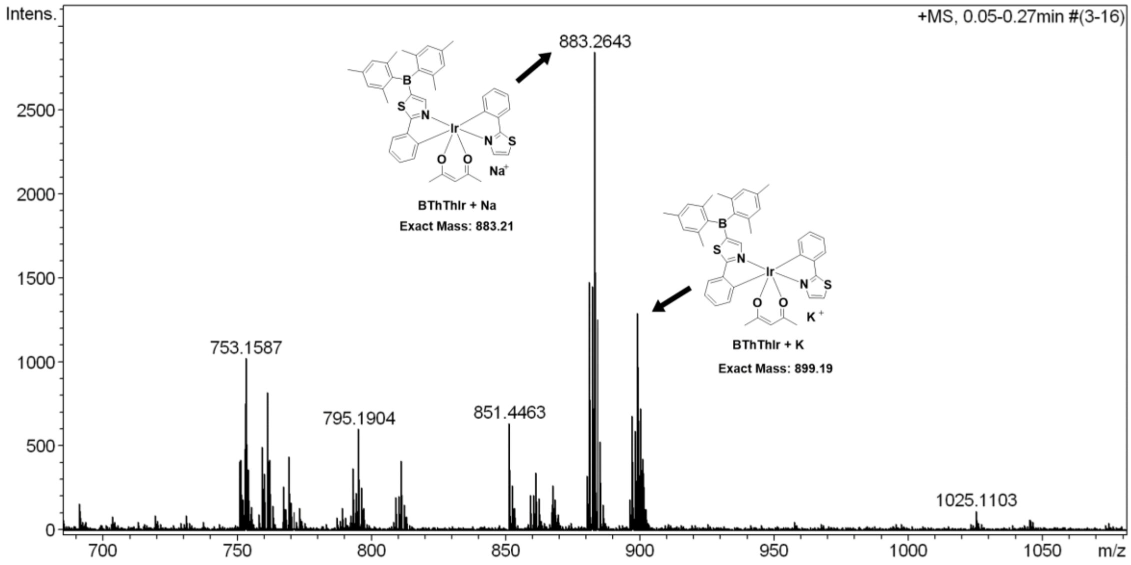
Intens.
+MS, 0.05-0.27min #(3-16)
883.2643
BThThIr + Na
Exact Mass: 883.21
BThThIr + K
Exact Mass: 899.19
753.1587
795.1904
851.4463
1025.1103
m/z

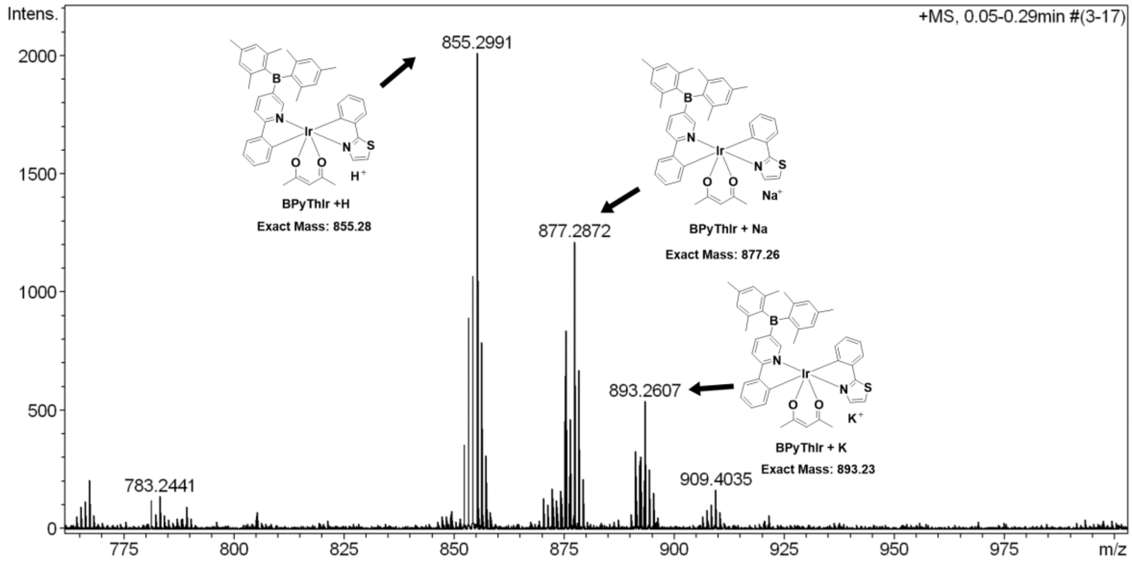
Intens.
+MS, 0.05-0.29min #(3-17)
855.2991
BPyThIr +H
Exact Mass: 855.28
877.2872
BPyThIr + Na
Exact Mass: 877.26
893.2607
BPyThIr + K
Exact Mass: 893.23
783.2441
909.4035
m/z

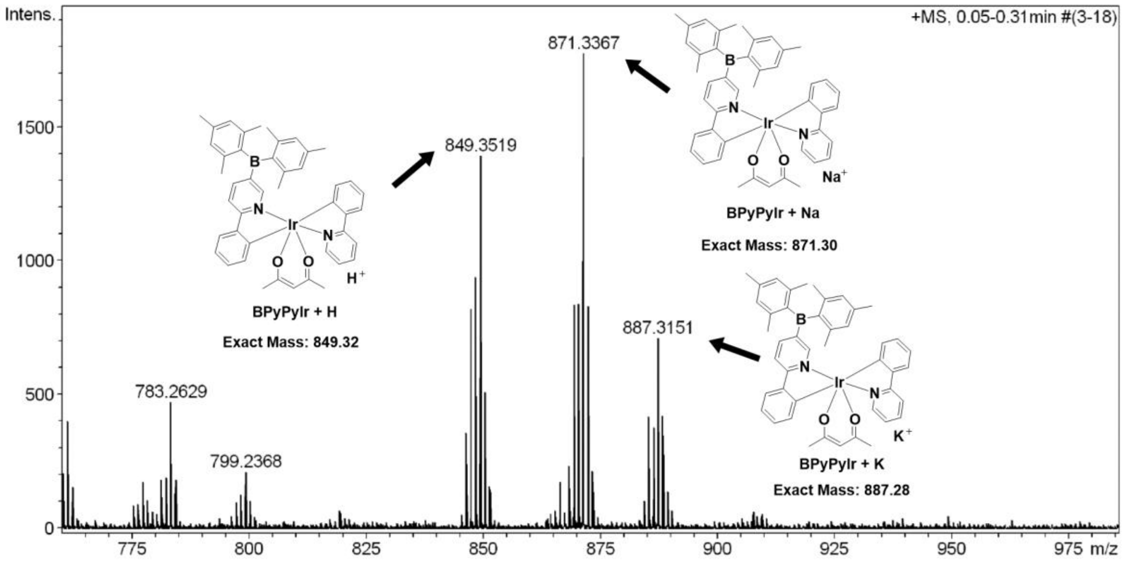
Intens.
+MS, 0.05-0.31min #(3-18)
871.3367
849.3519
BPyPyIr + H
Exact Mass: 849.32
BPyPyIr + Na
Exact Mass: 871.30
887.3151
BPyPyIr + K
Exact Mass: 887.28
783.2629
799.2368
m/z

Figure S2. MS spectra of **BThThIr**, **BPyThIr** and **BPyPyIr**.

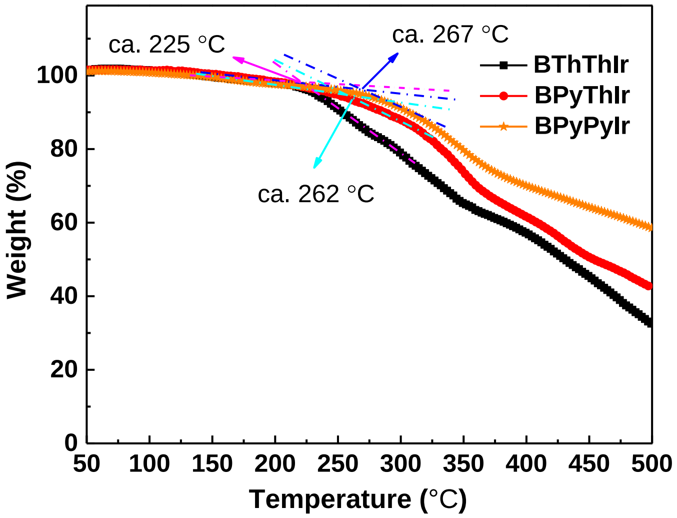

Figure S3. TGA curves recorded at a heating rate of 20 K min^{-1}.

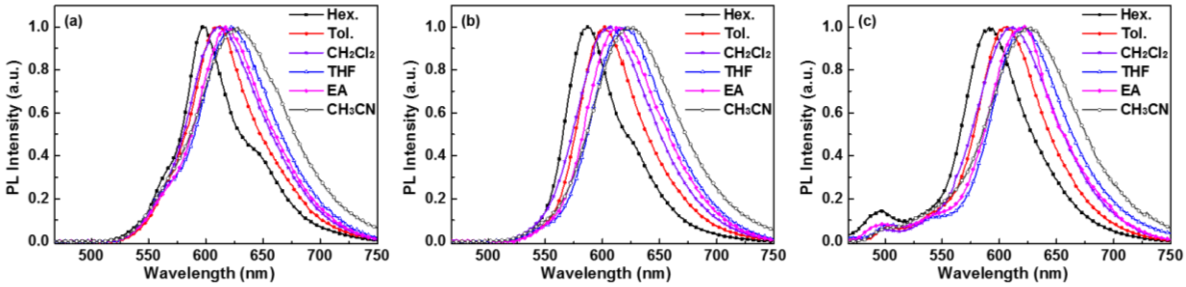

Figure S4. Emission spectra of (a) **BThThIr**, (b) **BPyThIr** and (c) **BPyPyIr** in various solvents at room temperature (**Hex.**, hexane; **Tol.**, toluene; **EA**, ethyl acetate).

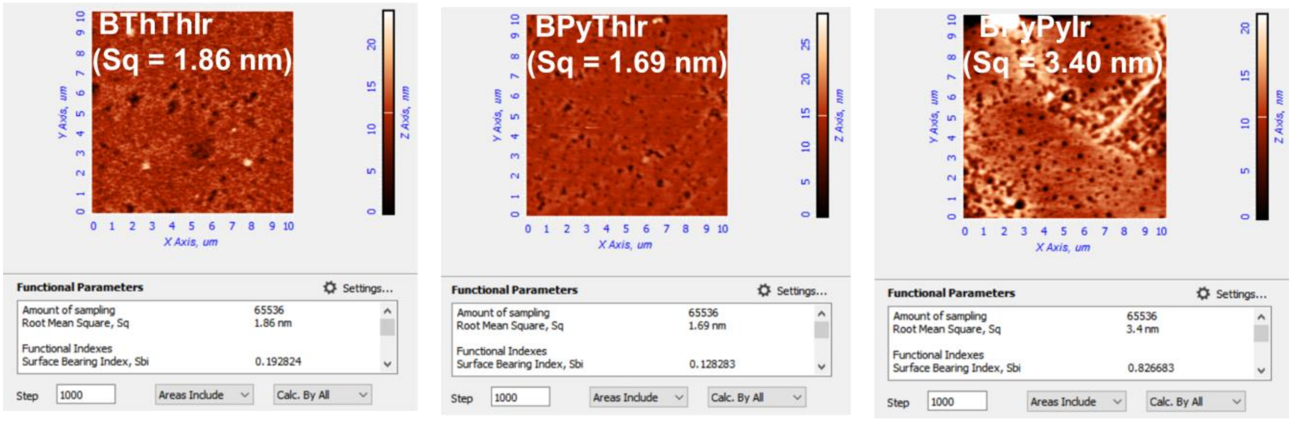

Figure S5. AFM topographic images of TCTA films doped with 10 wt% Ir(III) complexes.

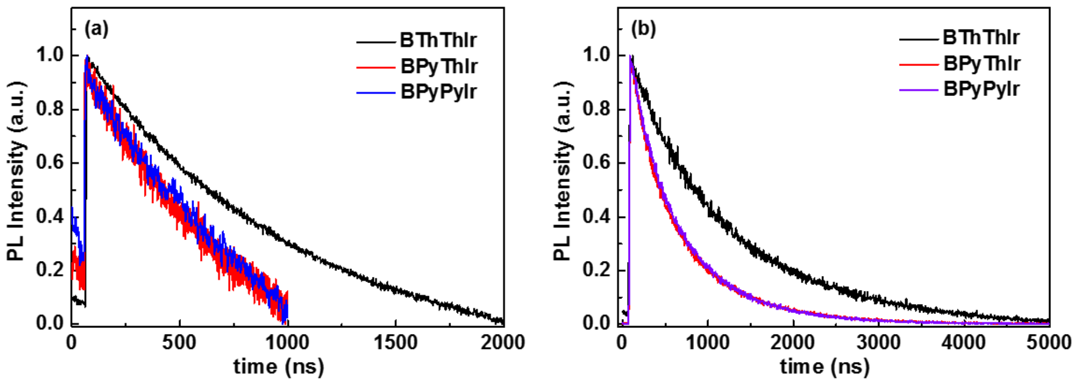

Figure S6. The transient PL decay curves of these Ir(III) complexes (a) in degassed THF solutions and (b) in doped TCTA films (at 10 wt%).

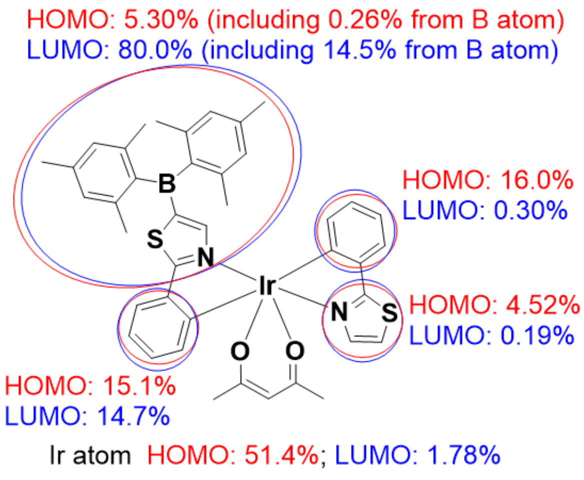

BThThIr

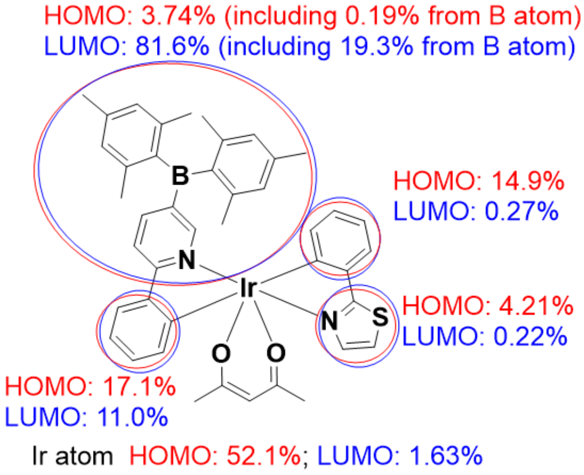

BPyThIr

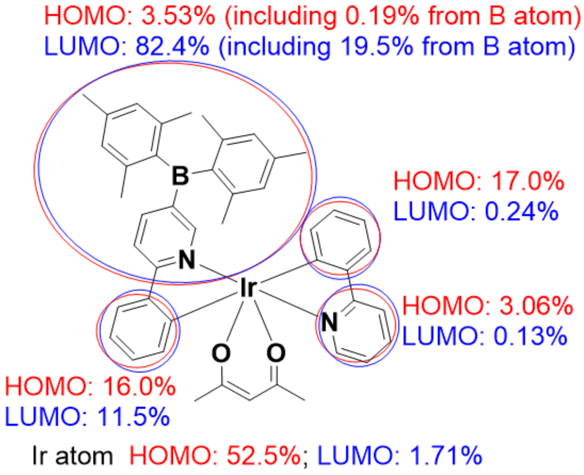

BPyPyIr

Figure S7. Contributions from each fragments in **BThThIr**, **BPyThIr** and **BPyPyIr** to HOMO and LUMO.

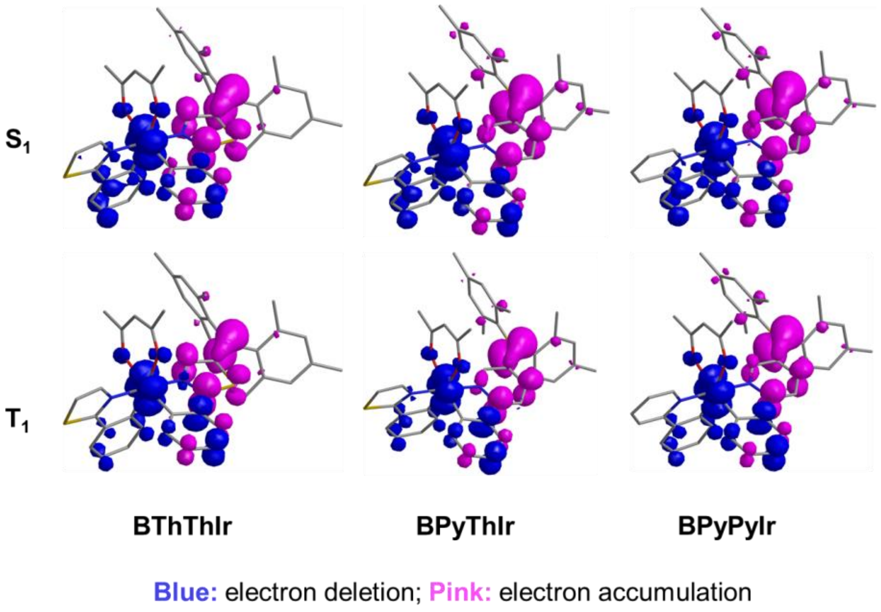

Figure S8. The plots of charge density difference (CDD) for both S_1 (top) and T_1 (bottom) excited states of these Ir(III) complexes.

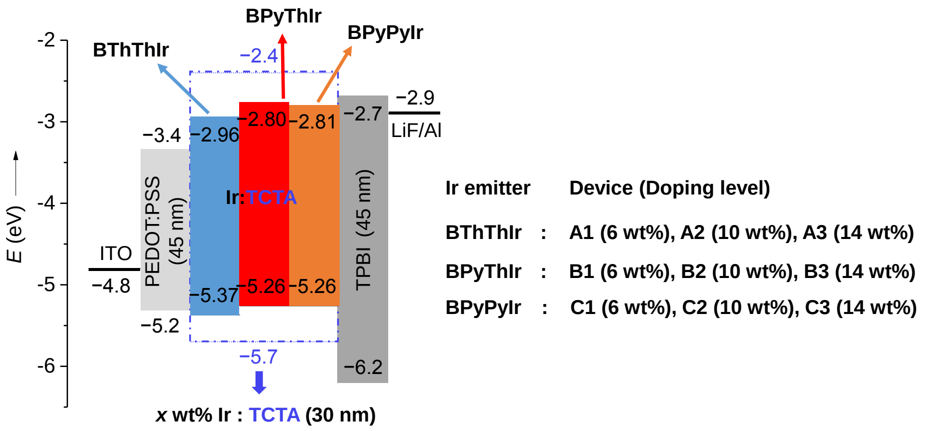

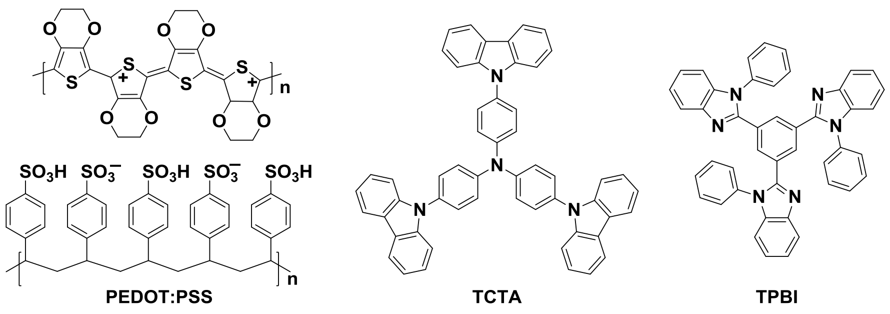

Figure S9. Energy levels and the molecular structures of the materials used in the fabricated OLEDs.

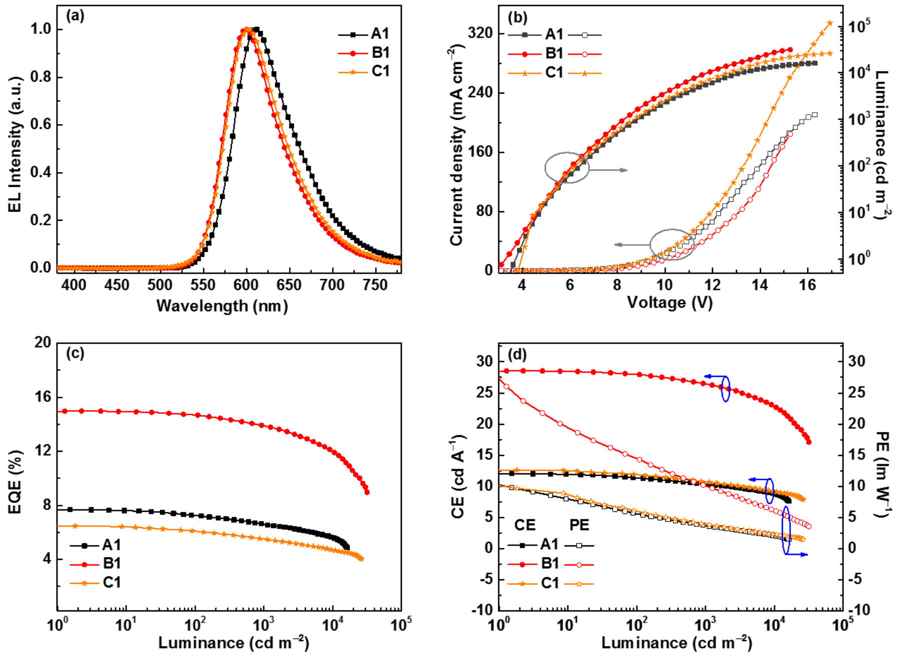

Figure S10. EL characteristics of devices **A1**, **B1** and **C1**: (a) EL spectral, (b) J – V – L characteristics, (c) curves of EQE vs luminance, and (d) curves of CE and PE vs luminance.

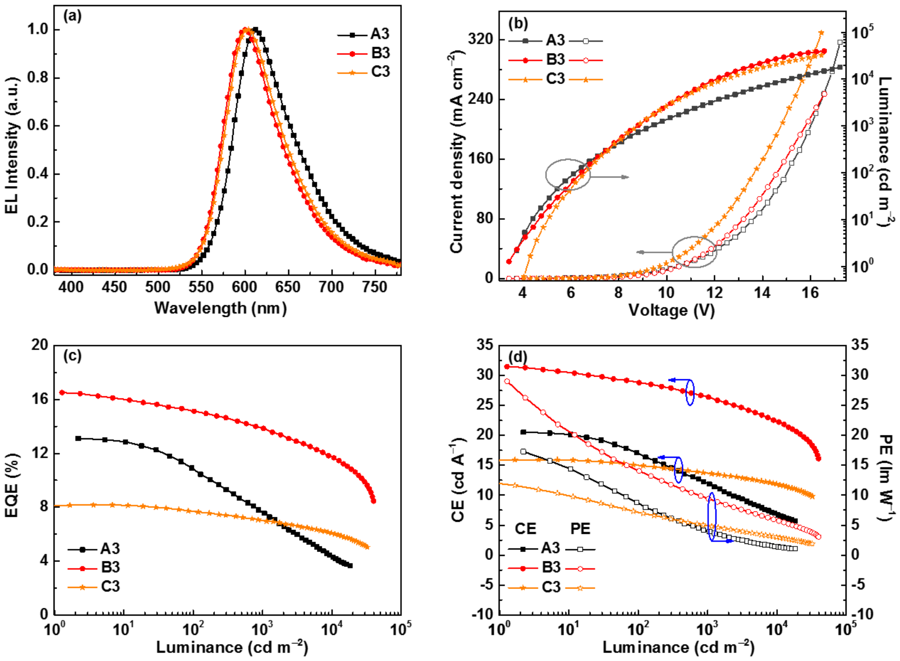

Figure S11. EL characteristics of devices **A3**, **B3** and **C3**: (a) EL spectral, (b) J – V – L characteristics, (c) curves of EQE vs luminance, and (d) curves of CE and PE vs luminance.

Table S1. EL data for device **A1**, **B1**, **C1**, **A3**, **B3** and **C3**.

	Emitter	λ_{EL}^{max} [nm]	V [V] [a)]	L_{max} [cd m^{-2}]	EQE [%] [b)]	CE [cd A^{-1}] [b)]	PE [lm W^{-1}] [b)]	CIE (x, y)
**A1**	**BThThIr** (6 wt%)	612	3.7/9.0/13.3	16048	7.7/7.3	12.0/10.3	11.4/3.6	(0.62, 0.38)
**B1**	**BPyThIr** (6 wt%)	604	3.4/8.2/11.8	31543	15.0/13.9	28.6/26.5	28.8/10.2	(0.61, 0.39)
**C1**	**BPyPyIr** (6 wt%)	604	3.8/8.6/12.8	26183	6.5/5.5	12.7/10.7	10.6/3.9	(0.60, 0.40)
**A3**	**BThThIr** (14 wt%)	612	3.6/9.4/15.0	18209	13.1/7.6	20.5/12.0	17.3/4.1	(0.62, 0.38)
**B3**	**BPyThIr** (14 wt%)	604	3.4/8.8/12.2	39866	16.5/13.9	31.5/26.4	29.0/9.4	(0.61, 0.39)
**C3**	**BPyPyIr** (14 wt%)	604	4.1/8.8/12.5	32302	8.2/7.0	15.9/13.7	12.3/4.9	(0.60, 0.40)

a) Driving voltages (V) in the order of at 1, 1000 and 10000 cd m^{-2}. b) EQE, CE and PE in the order of the maximum value and at 1000 cd m^{-2}.

References

[1] X. Yang, N. Sun, J. Dang, Z. Huang, C. Yao, X. Xu, C.-L. Ho, G. Zhou, D. Ma, X. Zhao, W.-Y. Wong, *J. Mater. Chem. C* **2013**, *1*, 3317.

[2] W. R.Wadt, P. J. Hay, *J. Chem. Phys.*, **1985**, *82*, 284.

[3] W. R.Wadt, P. J. Hay, *J. Chem. Phys.*, **1985**, *82*, 299.

[4] Gaussian 09, Revision A.02, M. J. Frisch, G. W. Trucks, H. B. Schlegel, G. E. Scuseria, M. A. Robb, J. R. Cheeseman, G. Scalmani, V. Barone, B. Mennucci, G. A. Petersson, H. Nakatsuji, M. Caricato, X. Li, H. P. Hratchian, A. F. Izmaylov, J. Bloino, G. Zheng, J. L. Sonnenberg, M. Hada, M. Ehara, K. Toyota, R. Fukuda, J. Hasegawa, M. Ishida, T. Nakajima, Y. Honda, O. Kitao, H. Nakai, T. Vreven, J. A. Montgomery, J. E. P. Jr., F. Ogliaro, M. Bearpark, J. J. Heyd, E. Brothers, K. N. Kudin, V. N. Staroverov, R. Kobayashi, J. Normand, K. Raghavachari, A. Rendell, J. C. Burant, S. S. Iyengar, J. Tomasi, M. Cossi, N. Rega, J. M. Millam, M. Klene,

J. E. Knox, J. B. Cross, V. Bakken, C. Adamo, J. Jaramillo, R. Gomperts, R. E. Stratmann, O. Yazyev, A. J. Austin, R. Cammi, C. Pomelli, J. W. Ochterski, R. L. Martin, K. Morokuma, V. G. Zakrzewski, G. A. Voth, P. Salvador, J. J. Dannenberg, S. Dapprich, A. D. Daniels, Ö. Farkas, J. B. Foresman, J. V. Ortiz, J. Cioslowski, D. J. Fox, Gaussian, Inc., Wallingford CT, **2009**.
